# Use of psychotropic medication and risk of road traffic crashes: a registry-based case–control study in Denmark, 1996–2018

**DOI:** 10.1007/s00213-022-06146-0

**Published:** 2022-04-23

**Authors:** Anne Vingaard Olesen, Tanja Kidholm Osmann Madsen, Harry Lahrmann, Jimmi Nielsen

**Affiliations:** 1 The Traffic Research Group, Department of the Built Environment, Aalborg University, Thomas Manns Vej 23, Aalborg Ø, 9220 Denmark; 2 The Mental Health Center Glostrup, Copenhagen University Hospital, Nordstjernevej 41, Glostrup, 2600 Denmark

**Keywords:** Psychotropic medication, Road traffic crash, Case–control study

## Abstract

**Rationale:**

Use of psychotropics is relatively prevalent amongst motor vehicle drivers because mobility is also important for persons suffering from psychiatric illness. However, medication side effects may increase the likelihood of being involved in traffic crashes.

**Objectives:**

This study aimed to assess the association between the use of four types of medication (antipsychotics, benzodiazepines and z-hypnotics, antidepressants and stimulants of ADHD treatment) and the risk of traffic crashes, in general, and single crashes subsequently.

**Method:**

We conducted a case–control study of data from 130,000 drivers involved in traffic crashes with personal injury and prescription data from all of Denmark during the period 1996–2018.

**Results:**

For antipsychotics, we found odds ratios of 0.86 and 1.29 for traffic crashes and single crashes, respectively; for benzodiazepines and z-hypnotics, 1.29 and 2.49, respectively; for antidepressants, 1.30 and 2.25, respectively; and for stimulants of ADHD treatment, 1.62 and 1.95, respectively. All *p* values were below 0.001.

**Conclusions:**

Based on our results on twofold increased risks of single crashes and moderately increased risks in persons with ADHD, it might seem tempting to ban psychotropic medication in traffic. Conversely, we accept increased risks of traffic crashes in young drivers and in the physically disabled with special aids and, to some extent, with exposure to alcohol. In the end, it is the authorities who must review the evidence and decide whether to prohibit (some types of) psychotropic medication in traffic. Finally, underlying disease and not the drug may increase the risk of being involved in a traffic crash.

## Introduction

Cognitive functioning is important for daily life tasks, such as driving a car. However, most psychiatric disorders are associated with poor cognitive functioning, such as fatigue and concentration problems. Furthermore, the lifetime prevalence of mental disorders is more than 30% (Pedersen et al. 2014). Brunnauer and colleagues conducted an exploratory study of the mobility behaviour of patients with psychiatric disorders and found that as many as 67% had a driving license, 77% of whom drove on a regular basis (Brunnauer et al. 2016). In a control group of neurological patients, the figures were 89% and 92%, respectively. Psychiatric patients who abstained or stopped driving were mainly female, of older age, those who drew a pension or who suffered from an organic mental disease or schizophrenic disorder (Brunnauer et al. 2016). Unfortunately, mental disorder is but one factor that potentially affects cognitive functioning; the psychotropics used to treat it may also deteriorate cognition. A decline in cognitive function from both mental illness and, potentially, the use of psychotropics may increase the risk of being involved in traffic crashes. Clinical studies indicate that psychotropic medication can help psychiatric patients drive a car safely if compliant with medication and are long-term medication users (Brunnauer et al. 2021). However, there may still be a core of severely ill patients who do not benefit from modern psychotropic medication in terms of their fitness to drive (Brunnauer et al. 2021).

For a long time, the risk of being involved in traffic crashes following the ingestion of benzodiazepines or other z-hypnotics has been considered high (Barbone et al. 1998). A roadside survey in relation to the DRUID (DRiving Under the Influence of Drugs) framework programme indicated that the prevalence of benzodiazepine use among drivers in Denmark was relatively low (0.47%) compared with other European countries, whereas the prevalence of z-drugs was high (0.32%) compared with other European countries (European Monitoring Centre for Drugs and Drug Addiction (EMCDDA) 2012). In current clinical practice in Denmark and other countries, there is a recommendation that the prescription of benzodiazepines and z-hypnotics for anxiety and restlessness ought to be accompanied by a driving prohibition (Hansen et al. 2014). Regarding the use of these drugs in the management of insomnia, the recommended maximum daily doses are provided by the national health authorities. Previous studies have found increased risk of considerable magnitude, but a meta-analysis from 2013 suggested a modest increased risk (17%) of involvement in traffic crashes with personal injury amongst users compared with non-users (Elvik 2013). To the best of our knowledge, no studies have investigated the effect of benzodiazepines and z-hypnotics or other psychotropics on the risk of a single traffic crash, defined as a crash involving only the user of psychotropics, with clear knowledge about their responsibility in the crash. Clinical studies have indicated that at least long-term users of benzodiazepines experience no impairment in on-road tests (Brunnauer et al. 2021).

In Denmark, as in many other countries, the decision on whether to ban driving is made by the prescriber of the medication (Hansen et al. 2014). Regarding the use of antipsychotics, antidepressants and other types of medication, the psychiatrist will distinguish between active prescription drugs to be taken before bedtime and those that should be taken at other times. Active substances ingested during daytime may immediately slow or tire those who use the drug before driving. Professional drivers are subjected to even stricter rules (Danish Patient Safety Authority 2017). Knowledge of the specific impact of antipsychotics on the risk of traffic crashes is very sparse, except for two case–control studies reporting no statistically significant association between road traffic crashes and antipsychotic use (Chang et al. 2013; Ravera et al. 2011). Clinical studies suggest that second-generation antipsychotics, in particular, can improve fitness to drive but also indicate that even under steady-state medication with antipsychotics, almost one-third (31%) of persons with schizophrenia or schizoaffective disorder show severe impairment of driving skills (Brunnauer et al. 2021).

The use of antidepressants seems to increase the risk of driver-involved in traffic crashes. However, in addition to antidepressant use, traffic crashes can also be attributed to depression itself as depressive symptoms impair cognitive functioning (Cameron and Rapoport 2016). A meta-analysis by Elvik suggested a 35% increased risk of traffic crashes involving personal injury from antidepressant use (Elvik 2013). Recent clinical studies have shown that modern antidepressants improve or at least stabilise the driving skills of persons with depression (Brunnauer et al. 2021; Brunnauer and Laux 2013, 2017).

Persons with attention deficit hyperactivity disorder (ADHD) experience various cognitive, emotional and social functioning impairments, which can have considerable consequences when driving (Fuermaier et al. 2017). However, evidence of the isolated effect of stimulants in ADHD treatment on the risk of traffic crashes remains sparse and divergent. Notably, a few clinical studies have suggested a beneficial effect on the fitness to drive of persons with ADHD (Cox et al. 2012; Sobanski et al. 2008, 2013).

The aim of this study is to quantify the risk of traffic crashes amongst users of psychotropics in a large Danish dataset comprising information on 130,000 drivers involved in traffic crashes involving personal injury between 1996 and 2018. Particularly in relation to ADHD treatment, we aimed to obtain new evidence on the effect of stimulant use on the risk of traffic crashes. As traffic crash causation can be multifactorial, we sought to perform separate analyses on single crashes, that is, where no other road users were involved and where the responsibility for the crash was clear. We aimed to use high statistical power to run analyses on separate drug types within the four main categories of benzodiazepines and z-hypnotics, antipsychotics, antidepressants and stimulants for the treatment of ADHD.

## Methods

This registry-based study was conducted between January 1, 1996, and December 31, 2018, with data obtained from all of Denmark, where a unique civil registration number is given to all Danes at birth (or immigration). This 10-digit number is used in most administrative registers, allowing the linkage of police-registered involvement in traffic crashes and prescription data.

We investigated the association between the use of psychotropics and traffic crash risk in a matched case–control design. The cases were identified as drivers (of cars, taxis, vans, trucks and buses) involved in traffic crashes involving personal injury and registered in police records. To become eligible for inclusion in the study, the drivers had to have a Danish civil registration number, thus resident in the country, and be old enough to hold a driver’s licence (18 years in the period 1996–2016; 17 years in the period 2017–2018). No assessment of responsibility was made by the police, but we ran separate analyses of single crashes (including collisions with objects, etc.) with the aim of assessing the use of psychotropics in crashes in which there was no doubt about the responsibility. Five controls were selected per case, who were individually matched by sex and age and alive and residing in Denmark on the crash date of the case. We selected the controls based on their residence in Denmark on January 1 of the crash year of the case. In order not to introduce skewness between the cases and controls, we removed those cases who were non-residents on January 1 of the crash year from the study population (approximately 1%). The controls were selected from the population registry because we could only obtain valid data from the Danish registry of driver’s licenses from 2018.

In Denmark, psychotropics are available by prescription only. Therefore, we used the Prescription Database to identify prescriptions for psychotropic medication filled by the study subjects during the study period. The Prescription Database contains data on the type of drug prescribed according to the al Therapeutic Chemical (ATC) classification system and the date the prescription was filled. We identified all the recorded prescriptions for benzodiazepines and z-hypnotics (ATC codes: N05BA, N05CD, N05CF and N05AE), antipsychotic medication (ATC code: N05A, excluding N05AN01), antidepressants (ATC code: N06A) and stimulants for ADHD treatment (ATC code: N06BA). We used an exposure window of 90 days before the crash date of the cases.

Statistical analyses were conducted using conditional logistic regression to estimate the odds ratio comparing the odds (or risk because of the low population risk of being involved in a traffic crash) between users and non-users. We performed both unadjusted analyses and analyses adjusted for marital status, socioeconomic status, taxable income and the highest educational level attained. The taxable income of all Danes above the age of 18 was divided into quartiles year-wise, and the study population was distributed into these quartiles. Individual data on the demographic variables were available through Statistics Denmark and linked to population registries using civil registration numbers.

A significance level of 0.05 was chosen. All statistical analyses were conducted using Stata 16 (StataCorp. 2019). Finally, we selected all psychotropic medications used by at least 200 cases and performed separate analyses of the associations with the risk of traffic crash. Furthermore, we added similar separate analyses of three commonly used antipsychotics, namely, olanzapine, risperidone and aripiprazole.

## Results

A total of 129,974 drivers involved in traffic crashes involving personal injury were included as cases in this study. Table 1 describes the case population with information from police records. Most car drivers were involved in crashes involving personal injury (81%), which corresponds well with the fact that, in 2018, road traffic in private cars in Denmark accounted for 79.8% of the total number of kilometres driven in four-wheeled motor vehicles (Statistics Denmark 2021). The road traffic shares of vans, taxis, trucks and buses were 14.0%, 0.8%, 4.2% and 1.3%, respectively, in 2018.

**Table 1:** Description of cases involved in police-registered traffic crashes with personal injury (*N* = 129,974)

	Cases	%
**Motor vehicle type**		
Car	105,643	81.28
Taxi	1918	1.48
Van	13,395	10.31
Truck	6,257	4.81
Bus	2761	2.12
**Driver’s license**		
Yes	125,238	96.36
Yes but not for the used vehicle type	491	0.38
No	4245	3.27
**Crash situation**		
Single crash	16,394	12.61
Rear-end collisions	20,983	16.14
Head-on collisions	16,397	12.62
Turning collisions, vehicles from the same direction	14,632	11.26
Turning collisions, vehicles from the opposite direction	13,512	10.40
Vehicles going straight from different roads	17,088	13.15
Vehicles from different roads, with at least one turning	17,022	13.10
Parked vehicles	1967	1.51
Pedestrian collisions	10,867	8.36
Collision with an animal, object, train etc	1112	0.86
**Driving under the influence of alcohol**		
Yes	16,413	12.63
No	113,561	87.37
**Crash year**		
1996–2000	42,896	33.00
2001–2005	35,347	27.20
2006–2010	25,780	19.83
2011–2015	16,500	12.69
2016–2018	9451	7.27

Almost all drivers in the crash cases held valid driver’s licences (96%). Regarding the crash situation, Table 1 shows a total of 17,506 (13%) single crashes and collisions with animals, objects, trains, etc., that is, crashes in which no other road users were involved. We call all these *single crashes*. Thirteen percent of the drivers involved in these crashes were driving under the influence of alcohol. Regarding the distribution of drivers in crashes by calendar period, there was a marked decrease from the beginning of the study period to the end, which is comparable to the figures in other European countries.

Table 2 shows that more controls than cases were married. In terms of taxable income, more controls were found to be below the first quartile and above the third quartile. A social gradient can be seen in Table 2, implying that the cases more frequently included employees at the basic level, other employees and employees not further specified. There were almost twice as many students amongst the controls than amongst the cases. Educational level was also higher amongst the controls, with one exception: there were slightly more cases with a vocational education and training background.

**Table 2:** Comparison of cases and controls

Variable	Cases %	Controls %
	*N* = 129,974	*N* = 649,870
**Sex**		
Male	71.67	71.67
Female	28.33	28.33
**Age**		
17–24	20.78	20.78
25–34	22.28	22.28
35–44	19.74	19.74
45–54	16.04	16.04
55–64	11.1	11.1
65–74	6.01	6.01
75–84	3.44	3.44
85 +	0.62	0.62
**Marital status**		
Married	41.71	43.63
Not married	58.29	56.37
**Taxable income**		
Below the first quartile	24.36	25.65
First to second quartiles	21.54	21.36
Second to third quartiles	27.38	25.29
Above the third quartile	26.72	27.71
**Socio-economic status**		
Self-employed with 10 or more employees	0.08	0.07
Self-employed with 5–9 employees	0.22	0.16
Self-employed with 1–4 employees	1.6	1.2
Self-employed with no employees	4.43	3.33
With an assisting spouse	0.16	0.16
Employees with management work	1.76	2.01
Employees in jobs that require skills at the highest level	5.55	8.04
Employees in jobs that require skills at the medium level	8.09	9.54
Employees in jobs that require skills at the basic level	30.87	26.67
Other employees	7.08	5.61
Employees not further specified	8.92	6.67
Unemployed	2.48	2.45
Temporarily outside the labour force (leave, sickness benefits, etc.)	1.88	1.31
Students	5.82	10.96
Old-age pensioners	8.33	8.7
Early retirement	5.22	6.77
Recipients of cash benefit	4.00	3.25
Other persons	3.48	3.06
Unknown status	0.01	0.04
**Education**		
Primary education	39.8	33.35
Upper secondary education	6.28	10.2
Vocational education and training	34.21	32.4
Short-cycle higher education	3.06	3.48
Vocational bachelor’s education	8.6	9.63
Bachelor’s degree	0.6	1.39
Master’s degree	3.66	5.38
PhD degree	0.15	0.31
Unknown education	3.65	3.86

Table 3 shows that the risk of a traffic crash involving personal injury increased by 24% for users of any type of psychotropics compared with non-users. Similarly, the pooled risk of a single crash was elevated by 117% for users compared with non-users. For antipsychotics, the adjusted analysis showed that a 14% decreased risk of involvement in any traffic crash involving personal injury was clearly statistically significant because of the high statistical power. However, when the analysis was restricted to single crashes, the adjusted risk was estimated at an increase of 29% in comparison between users and non-users. The use of antidepressants and benzodiazepines/z-hypnotics was more frequent than that of antipsychotics, and the adjusted risk of involvement in traffic crashes involving personal injury, in general, was approximately 30% higher in users than in non-users of the two types of medication. The adjusted risk of an at-fault single crash was 125% and 149% higher for users than for non-users of antidepressants and benzodiazepines/z-hypnotics, respectively. Users of stimulants for the treatment of ADHD faced a 62% increased risk of traffic crashes, in general, with the risk of a single crash being almost doubled that for non-users.

**Table 3:** Odds ratio estimates of being involved in a traffic crash with personal injury as a driver of a motor vehicle: comparison of users of psychotropics with non-users

				Unadjusted			Adjusted*		
Medication type	N cases	% exposed cases	% exposed controls	OR	95% CI	*p* value	OR	95% CI	*p* value
**Antipsychotics**									
Traffic crashes, in general	129,974	1.03	1.26	0.81	0.77–0.86	< 0.001	0.86	0.81–0.91	< 0.001
Single crashes in which the fault is clear	17,506	1.92	0.95	2.04	1.79–2.32	< 0.001	1.29	1.13–1.48	< 0.001
**Antidepressants**									
Traffic crashes, in general	129,974	4.46	3.55	1.28	1.24–1.31	< 0.001	1.30	1.26–1.34	< 0.001
Single crashes in which the fault is clear	17,506	6.75	2.62	2.77	2.57–2.98	< 0.001	2.25	2.08–2.43	< 0.001
**Benzodiazepines + z-hypnotics**									
Traffic crashes, in general	129,974	4.76	3.90	1.25	1.21–1.28	< 0.001	1.29	1.25–1.33	< 0.001
Single crashes in which the fault is clear	17,506	7.05	2.47	3.26	3.03–3.52	< 0.001	2.49	2.29–2.70	< 0.001
**ADHD medication stimulants**									
Traffic crashes, in general	129,974	0.28	0.16	1.83	1.62–2.06	< 0.001	1.62	1.43–1.83	< 0.001
Single crashes in which the fault is clear	17,506	0.63	0.20	3.14	2.47–4.00	< 0.001	1.95	1.51–2.51	< 0.001
**Any of the four types of medication**									
Traffic crashes, in general	129,974	8.47	7.17	1.21	1.18–1.24	< 0.001	1.24	1.21–1.27	< 0.001
Single crashes in which the fault is clear	17,506	12.38	5.09	2.81	2.65–2.97	< 0.001	2.17	2.05–2.32	< 0.001

*Adjustment for marital status, income, socio-economic status and education. Controls matched by sex and age

Table 4 shows separate analyses of 22 psychotropic drugs with more than 200 users amongst the cases (percentage of exposed cases higher than 0.15) supplemented by analyses of three frequently used antipsychotics (olanzapine, risperidone and aripiprazole). Most of the 22 drugs were associated with an increased risk of less than 30%, whereas one group of drugs (zolpidem, venlafaxine, nitrazepam, escitalopram and mianserin) reached a moderate risk level of around 50%. Two drugs (chlordiazepoxide and methylphenidate) were associated with increased risks of 74% and 67%, respectively, of any type of traffic crash involving personal injury. Regarding the three antipsychotics, we observe statistically significantly reduced risks of involvement in traffic crashes involving personal injury in concordance with the results presented in Table 3 regarding antipsychotics.

**Table 4:** Analyses of single psychotropic medication types most common amongst the cases in terms of risk of traffic crashes with personal injury plus olanzapine, risperidone and aripiprazole

				Unadjusted			Adjusted*		
ATC code	Type	% exposed cases	% exposed controls	OR	95% CI	*p* value	OR	95% CI	*p* value
N06AB04	Citalopram	1.46	1.17	1.25	1.19–1.32	< 0.001	1.26	1.20–1.33	< 0.001
N05CF01	Zopiclone	1.17	0.96	1.22	1.15–1.29	< 0.001	1.25	1.18–1.33	< 0.001
N05BA01	Diazepam	0.94	0.77	1.22	1.15–1.30	< 0.001	1.27	1.19–1.36	< 0.001
N05CF02	Zolpidem	0.88	0.61	1.46	1.36–1.56	< 0.001	1.50	1.40–1.60	< 0.001
N05BA04	Oxazepam	0.76	0.63	1.21	1.13–1.30	< 0.001	1.24	1.16–1.33	< 0.001
N06AX16	Venlafaxine	0.56	0.36	1.58	1.45–1.72	< 0.001	1.57	1.45–1.71	< 0.001
N06AB06	Sertraline	0.56	0.47	1.21	1.12–1.31	< 0.001	1.21	1.12–1.32	< 0.001
N06AX11	Mirtazapine	0.52	0.41	1.27	1.17–1.38	< 0.001	1.27	1.16–1.38	< 0.001
N05CD02	Nitrazepam	0.49	0.35	1.41	1.29–1.54	< 0.001	1.46	1.33–1.59	< 0.001
N05BA12	Alprazolam	0.46	0.36	1.27	1.16–1.39	< 0.001	1.28	1.17–1.40	< 0.001
N06AB10	Escitalopram	0.32	0.23	1.37	1.23–1.53	< 0.001	1.40	1.25–1.56	< 0.001
N06AB05	Paroxetine	0.31	0.24	1.32	1.18–1.47	< 0.001	1.31	1.17–1.46	< 0.001
N06AA09	Amitriptyline	0.27	0.24	1.13	1.00–1.26	0.045	1.17	1.04–1.31	0.01
N06AB03	Fluoxetine	0.27	0.22	1.24	1.10–1.39	< 0.001	1.26	1.12–1.42	< 0.001
N05AH04	Quetiapine	0.23	0.18	1.26	1.11–1.43	< 0.001	1.25	1.10–1.43	0.001
N05AF03	Chlorprothixine	0.23	0.20	1.17	1.03–1.33	0.014	1.23	1.08–1.40	0.002
N05BA08	Bromazepam	0.23	0.19	1.18	1.04–1.34	0.009	1.21	1.07–1.38	0.003
N06BA04	Methylphenidate	0.23	0.12	1.89	1.65–2.16	< 0.001	1.67	1.45–1.91	< 0.001
N05BA02	Chlordiazepoxide	0.20	0.11	1.78	1.55–2.05	< 0.001	1.74	1.51–2.01	< 0.001
N06AX03	Mianserin	0.20	0.14	1.42	1.24–1.63	< 0.001	1.45	1.24–1.66	< 0.001
N05CD05	Triazolam	0.19	0.16	1.21	1.05–1.39	0.007	1.24	1.08–1.43	0.002
N05AA02	Levomepromazine	0.16	0.18	0.90	0.78–1.05	0.17	0.97	0.84–1.13	0.692
N05AH03	Olanzapine	0.13	0.19	0.71	0.61–0.83	< 0.001	0.80	0.68–0.93	0.005
N05AX08	Risperidone	0.08	0.14	0.56	0.46–0.68	< 0.001	0.61	0.50–0.75	< 0.001
N05AX12	Aripiprazole	0.04	0.05	0.67	0.49–0.91	0.01	0.69	0.51–0.95	0.02

*Adjustment for marital status, taxable income, socio-economic status and education. Controls matched by sex and age

## Discussion

This study found that the risk of a single traffic crash; that is, a crash involving no other road user was at least doubled amongst users of antidepressants, benzodiazepines and z-hypnotics and stimulants for the treatment of ADHD. Regarding traffic crashes involving personal injury, in general (both single and multiparty crashes), we found only slightly increased risks amongst drivers under the influence of psychotropic medication. This excluded users of stimulants for the treatment of ADHD, for whom the risk of traffic crash involvement increased by a moderate 62% compared with non-users. For antipsychotics, we found a protective effect on traffic crashes, in general. Further studies are needed to explain this finding.

These results raise at least two questions, one of which deals with the difference between the doubled risk of single crashes compared with the slightly increased risk of involvement in crashes in general. The second question relates to the magnitude of the increased risks. Regarding the first question, we argue that the risk associated with the use of psychotropic medication was attenuated when several road users were involved in a crash. Single crashes were included in the general odds ratio estimate but only accounted for 13.5% of the total number of drivers included in the general analysis. Another explanation for the higher risk of single crashes may be that they resulted from suicide attempts, for example driving against a tree, thus avoiding the increased risk of injury in other road users. Regarding the second question of whether the doubling of the risk of single crashes was large and should be a cause for concern, a comparison with other risk differences in traffic becomes relevant. For instance, the risk of fatal or severe injuries amongst 18–19-year-old drivers is seven times higher than amongst 55–64-year-old drivers, and for 20–24-year-old drivers, it is 4.5 times higher (Christiansen and Warnecke 2018). Similarly, driving with an alcohol concentration level of 0.5–0.8 g/L, that is, just above the legal blood alcohol concentration limit in Denmark, implies a risk of severe injury that was approximately four times higher than when driving sober (Hels et al. 2013). Driving with an alcohol concentration of 0.1–0.5 g/L is associated with a statistically insignificantly increased risk of severe injury of 30% (Hels et al. 2013). A third analogy, which we also tend to accept, stems from the likelihood of an elevated crash risk associated with physically disabled drivers operating their car with special aids. Summing up, we have provided three examples of cases (besides users of psychotropic drugs) associated with *acceptable* levels of increased risk of traffic crashes. However, it is relatively simple to eliminate the twofold increased risks of single crashes and moderately increased risks of traffic crashes, in general, in persons with ADHD. It is up to the authorities to weigh the evidence for and against a simple driving prohibition, which will affect the mobility of many citizens.

As mentioned above, the finding of a positive effect found in the use of antipsychotics requires further discussion. We found that the risk of traffic crashes generally was decreased by 14% in users of antipsychotics compared with non-users (with similar findings for olanzapine, risperidone and aripiprazole), whereas the risk of single crashes increased by 29%. All associations were statistically significant. There are two possible explanations for these findings: driving prohibition and refrain amongst users of antipsychotics, and those who drove did so at a lower rate than the average non-user. In both cases, users of antipsychotics were *protected* from involvement as drivers in traffic crashes because of their reduced driving exposure. Conversely, the risk of being involved in a single crash increased by 29% amongst users of antipsychotics, which seems contradictory considering the above-mentioned arguments regarding lower mileage and driving refrain. Therefore, why is there an increased risk of single crashes in users compared with non-users if users drive less and/or refrain from driving? Another explanation for these findings could be that the odds ratios estimated in a case–control design with matches for only gender and age did not necessarily match for controls holding a driver’s licence. We argue that the odds ratios may have been under-estimated because most of the controls did not hold a driver’s licence and were, thus, not at risk of being involved as drivers in traffic crashes unless they had chosen to drive without a licence. We had available data on all Danes with driver’s licences in 2018; additional analyses addressing this possible bias were performed, and a limited effect was found. However, the statistical power was low, so we did not pursue this issue further, including in relation to the other types of psychotropic medication.

The protective effect of antipsychotics could not be confirmed in the literature, perhaps because of the low power of other studies. Although Chang and colleagues generally found no association between road traffic crashes and antipsychotic prescriptions, they noted a statistically significantly increased risk of 60% for anxiolytics, which could be benzodiazepines and z-hypnotics, antidepressants and antipsychotics (Chang et al. 2013). The case–control study by Ravera and colleagues (2011) also found no statistically significant association between road traffic crashes and the use of antipsychotics, but the authors reported a 54% increased risk in users of anxiolytics (Ravera et al. 2011). However, as described by Brunnauer and colleagues, schizophrenia (and, thus, in many cases, treatment with antipsychotics) is predictive of driving cessation, potentially explaining our finding (Brunnauer et al. 2016).

Our study found a 29% increased risk of traffic crashes involving personal injury associated with the use of benzodiazepines and z-hypnotics, which is similar in magnitude to the result of a previous meta-analysis from 2013, indicating that the odds were increased by a modest 17% after adjustment for publication bias in the analysis of 51 effect estimates (Elvik 2013). A more recent Taiwanese study by Chang and colleagues found 56% and 42% increased risks of being involved in traffic crashes for persons who had filled a prescription of benzodiazepines and z-hypnotics, respectively (Chang et al. 2013). In an investigation of the association between sustaining a severe traffic injury and the use of benzodiazepines and z-hypnotics, Hels and colleagues found an odds ratio of 1.77 (Hels et al. 2013).

We found that drivers who used benzodiazepines and/or z-hypnotics had a 150% increased risk of ending up in a single crash. A French study found no association between being responsible for traffic crashes and the use of zopiclone and zolpidem (Orriols et al. 2011). Orriols and colleagues also estimated increased odds of 42% of being at fault in a traffic crash amongst users of benzodiazepines and z-hypnotics (Orriols et al. 2016).

Our result of an increased risk of 30% of being involved in a traffic crash amongst users of antidepressants compared with non-users is consistent with previous findings. In 2013, Elvik performed a meta-analysis of the effect of antidepressant use, in general, on the risk of traffic injuries and found that the odds (risks) increased by 35% after controlling for publication bias (Elvik 2013). Included in the meta-analysis by Elvik was the Norwegian registry study by Bramness and colleagues, which, along with Elvik and our study, found only slightly increased risks of being involved in a traffic crash after having filled a prescription for antidepressants (Bramness et al. 2008). Chang et al. found increased risks of 73%, 72% and 77% for antidepressants, in general, SSRIs and tricyclic antidepressants, respectively (Chang et al. 2013). Rapoport and colleagues conducted a cohort study of the effect of both first- and second-generation antidepressants on older drivers above the age of 65 years and found no association with the at-fault risk of traffic crashes for first-generation antidepressants but an increased risk of 10% for second-generation antidepressants (Rapoport et al. 2011). Cameron and Rapoport (2016) conducted a systematic review of the effect of antidepressants on driving amongst older persons above the age of 55 years and found an increased risk of involvement in crashes. The authors concluded that underlying depression was the culprit and not the medication itself (Cameron and Rapoport 2016). Furthermore, Aduen and colleagues showed that treatment with antidepressants attenuated the risk of crashes and near-crashes (Aduen et al. 2018).

We found a doubled risk of single crashes with clear responsibility. In general, however, Orriols and colleagues in 2013 showed increased odds of 34% of being responsible for a traffic crash in those who had filled a prescription for antidepressants (Orriols et al. 2012).

Our study also found moderately increased risks of traffic crashes amongst those who had filled prescriptions for stimulants for the treatment of ADHD. Chang et al. registered occurrences of motor vehicle crashes in a large cohort of persons with an ADHD diagnosis and compared medicated periods with un-medicated periods within the same individuals. They found that ADHD medication reduced the risk of motor vehicle crashes by approximately 40% (Chang et al. 2017). A Canadian study by Vingilis et al. from 2014 showed no association between self-reported use of ADHD medication and the self-reported motor vehicle collisions; however, their study sample was limited (Vingilis et al. 2014). Furthermore, Aduen and colleagues (2018) showed that treatment with stimulants did not attenuate the risk of crashes and near-crashes.

A social gradient in the occurrence of traffic crashes is evident from Table 2, which mean that the cases involved in traffic crashes were worse off socio-economically and educationally. This is a well-known phenomenon in road traffic safety—that drivers with lower levels of education tend to take more risks and drive in vehicles deemed less safe (Kruse 2015; Van den Berghe 2017). In relation to Table 1, we noted that the distribution of shares by motor vehicle type did not fully correspond with the distribution of road traffic in 2018. However, we were unable to provide a reasonable explanation for these discrepancies.

This case–control study was nationwide and complete, comprising data on almost 130,000 drivers in traffic crashes involving personal injury, which enabled us to address our hypotheses with high statistical power. The study linked individual police records of involvement in traffic crashes with prescription information at the individual level by using drivers’ unique personal identification numbers in Denmark.

One drawback of the study was that we could not sample controls holding a driver’s license. We only had valid data from the Danish driver’s license registry from 2018, which we used for the sensitivity analysis described in the discussion above. The Danish registry of driver’s licenses is a “living” registry; that is, the identification numbers of the deceased are expunged after a maximum of 2 years following their death, and their previous status as of a licensed driver is overwritten when the changes occur.

We could not adjust for mileage, which would probably imply a reduction in the odds ratio estimate for those who use stimulants for the treatment of ADHD because they would have likely driven more than the matched controls (Vaa 2014). One would also expect that those on other types of medication would drive less than the controls, implying that an adjustment for mileage would increase the odds ratio estimates.

The decrease in the number of drivers involved in crashes from the beginning of the study period to the end could be due to a combination of regular decreases in the number of crashes involving motor vehicles during the period and an increased degree of police underreporting. Adjustments were made for the calendar year and, thus, the trend of underreporting.

The study was based on filled prescriptions, so we could not be sure that the drugs were, in fact, ingested. This is the general condition under which all studies on the topic are conducted. In the event that those filling their prescriptions did not take the active drug, we would have overestimated the effect of serious psychiatric illness on the ability to drive safely.

Furthermore, there may have been confounding by indication. The users of some drugs may have been suffering from a particularly severe underlying disorder, in which cases, the underlying disease, and not the drug, may have increased the risk of being involved in a car crash (in the case of benzodiazepines and z-drugs, no underlying disorder needs to be present (abuse)). The simple case–control design cannot disentangle the effects of psychosis, depression and ADHD and the potentially impairing effects of their treatment. In order to disentangle clinical patient cohort studies, preferably randomised controlled trials must be conducted, for example, those done by Brunnauer and colleagues for specific antidepressants (Brunnauer et al. 2008, 2015; Brunnauer and Laux 2017). Even though the cited clinical studies demonstrated a clearly increased fitness to drive following a relatively short treatment period, the depression patients did not reach the level of driving skills of the healthy controls. Therefore, the authors concluded that psychomotor and cognitive impairment may have persisted after remission (Brunnauer et al. 2008). Similarly, other clinical studies involving patients with ADHD have investigated the impact of, for example, methylphenidate and found evidence that stimulants for the treatment of ADHD improved the skills needed to drive safely (Cox et al. 2012; Sobanski et al. 2008, 2013).

As stated earlier, the study shows that the use of psychotropic medication is associated with an increased risk of traffic crashes, but whether these results should lead to driving bans is, as in many other situations in road traffic, a political trade-off between the risk of crashes and mobility.

## References

[CR1] Aduen PA, Kofler MJ, Sarver DE, Wells EL, Soto EF, Cox DJ (2018). ADHD, depression, and motor vehicle crashes: a prospective cohort study of continuously-monitored, real-world driving. J Psychiatr Res.

[CR2] Barbone F, McMahon AD, Davey PG, Morris AD, Reid IC, McDevitt DG, MacDonald TM (1998). Association of road-traffic accidents with benzodiazepine use. Lancet.

[CR3] Bramness JG, Skurtveit S, Neutel CI, Mørland J, Engeland A (2008). Minor increase in risk of road traffic accidents after prescriptions of antidepressants: a study of population registry data in Norway. J Clin Psychiatry.

[CR4] Brunnauer A, Laux G (2013). The effects of most commonly prescribed second generation antidepressants on driving ability: a systematic review: 70th birthday Prof. Riederer J Neural Transm.

[CR5] Brunnauer A, Laux G (2017). Driving under the influence of antidepressants: a systematic review and update of the evidence of experimental and controlled clinical studies. Pharmacopsychiatry.

[CR6] Brunnauer A, Laux G, David I, Fric M, Hermisson I, Möller HJ (2008). The impact of Reboxetine and Mirtazapine on driving simulator performance and psychomotor function in depressed patients. J Clin Psychiatry.

[CR7] Brunnauer AV, Buschert MF, Distler G, Sander K, Segmiller F, Zwanzger P, Laux G (2015). Driving performance and psychomotor function in depressed patients treated with Agomelatine or Venlafaxine. Pharmacopsychiatry.

[CR8] Brunnauer A, Buschert V, Segmiller F, Zwick S, Bufler J, Schmauss M, Messer T, Möller HJ, Frommberger U, Bartl H, Steinberg R, Laux G (2016). Mobility behaviour and driving status of patients with mental disorders - an exploratory study. Int J Psychiatry Clin Pract.

[CR9] Brunnauer A, Herpich F, Zwanzger P, Laux G (2021). Driving performance under treatment of most frequently prescribed drugs for mental disorders: a systematic review of patient studies. Int J Neuropsychopharmacol.

[CR10] Cameron DH, Rapoport MJ (2016). Antidepressants and driving in older adults: a systematic review. Can J Aging.

[CR11] Chang CM, Wu ECH, Chen CY, Wu KY, Liang HY, Chau YL, Wu CS, Lin KM, Tsai HJ (2013). Psychotropic drugs and risk of motor vehicle accidents: a population-based case-control study. Br J Clin Pharmacol.

[CR12] Chang Z, Quinn PD, Hur K, Gibbons RD, Sjölander A, Larsson H, D’Onofrio BM (2017). Association between medication use for attention-deficit/hyperactivity disorder and risk of motor vehicle crashes. JAMA Psychiatry.

[CR13] Christiansen H, Warnecke ML (2018) Risiko i trafikken 2007–16 [Risk in traffic 2007–2016]. https://backend.orbit.dtu.dk/ws/portalfiles/portal/146185353/Risiko_i_trafikken_2007_16.pdf. Accessed 30 June 2021

[CR14] Cox DJ, Davis M, Mikami AY, Singh H, Merkel RL, Burket R (2012). Long-acting methylphenidate reduces collision rates of young adult drivers with Attention-Deficit/Hyperactivity Disorder. J Clin Psychopharmacol.

[CR15] Danish Patient Safety Authority (2017) Vejledning om helbredskrav til kørekort [Guidance to health requirements of licensed drivers]. https://stps.dk/da/udgivelser/2017/vejledning-om-helbredskrav-til-koerekort/~/media/9BE267FAC6AE4BE3ABB93FAA6E7C2347.ashx. Accessed 7 September 2021

[CR16] Elvik R (2013). Risk of road accident associated with the use of drugs: a systematic review and meta-analysis of evidence from epidemiological studies. Accid Anal Prev.

[CR17] European Monitoring Centre for Drugs and Drug Addiction (EMCDDA) (2012) Driving under the influence of drugs, alcohol and medicines in Europe - findings from the DRUID project. Luxembourg. https://www.emcdda.europa.eu/system/files/publications/743/TDXA12006ENN_402402.pdf. Accessed 19 March 2022.

[CR18] Fuermaier ABM, Tucha L, Evans BL, Koerts J, de Waard D, Brookhuis K, Aschenbrenner S, Thome J, Lange KW, Tucha O (2017). Driving and attention deficit hyperactivity disorder. J Neural Transm.

[CR19] Hansen HL, Grønlykke T, Fredsted B, Bryld C, Adelhardt M, Garsdal L, Danish Health Authority (2014) Traffic and medications. https://www.sst.dk/da/udgivelser/2014/rationel-farmakoterapi-2-2014/trafik-og-laegemidler. Accessed 7 October 2018

[CR20] Hels T, Lyckegaard A, Simonsen KW, Steentoft A, Bernhoft IM (2013). Risk of severe driver injury by driving with psychoactive substances. Accid Anal Prev.

[CR21] Kruse M (2015). Costs of traffic injuries. Inj Prev.

[CR22] Orriols L, Philip P, Moore N, Castot A, Gadegbeku B, Delorme B, Mallaret M, Largarde E (2011). Benzodiazepine-like hypnotics and the associated risk of road traffic accidents. Clin Pharmacol Ther.

[CR23] Orriols L, Queinec R, Philip P, Gadegbeku B, Delorme B, Moore N, Suissa S, Lagarde E (2012). Risk of injurious road traffic crash after prescription of antidepressants. J Clin Psychiatry.

[CR24] Orriols L, Luxcey A, Contrand B, Gadegbeku B, Delorme B, Tricotel A, Moore N, Salmi LR, Lagarde E (2016). Road traffic crash risk associated with benzodiazepine and z-hypnotic use after implementation of a colour-graded pictogram: a responsibility study. Br J Clin Pharmacol.

[CR25] Pedersen CB, Mors O, Bertelsen A, Waltoft BL, Agerbo E, McGrath JJ, Mortensen PB, Eaton WW (2014). A comprehensive nationwide study of the incidence rate and lifetime risk for treated mental disorders. JAMA Psychiatry.

[CR26] Rapoport MJ, Zagorski B, Seitz D, Herrmann N, Molnar F, Redelmeier DA (2011). At-fault motor vehicle crash risk in elderly patients treated with antidepressants. Am J Geriatr Psychiatry.

[CR27] Ravera S, van Rein N, de Gier JJ, de Jong-van den Berg LTW,  (2011). Road traffic accidents and psychotropic medication use in the Netherlands: a case-control study. Br J Clin Pharmacol.

[CR28] Sobanski E, Sabljic D, Alm B, Skopp G, Kettler N, Mattern R, Strohbeck-Kühner P (2008). Driving-related risks and impact of Methylphenidate treatment on driving in adults with Attention-Deficit/Hyperactivity Disorder (ADHD). J Neural Transm.

[CR29] Sobanski E, Sabljic D, Alm B, Dittmann RW, Wehmeier PM, Skopp G, Strohbeck-Kühner P (2013). Driving performance in adults with ADHD: results from a randomized, waiting list controlled trial with Atomoxetine”. Eur Psychiatry.

[CR30] StataCorp. (2019) STATA statistical software: Release 16

[CR31] Statistics Denmark (2021) Road traffic of Danish vehicles on Danish roads by means of transport. https://statistikbanken.dk/statbank5a/default.asp?w=1920. Accessed 30 June 2021

[CR32] Vaa T (2014). ADHD and relative risk of accidents in road traffic: a meta-analysis. Accid Anal Prev.

[CR33] Van den Berghe W (2017) The association between road safety and socioeconomic situation (SES). An international literature review. Vias Institute - Knowledge Centre Road Safety, Brussels, Belgium. https://www.vias.be/publications/HetverbandtussenSESenverkeersveiligheid/The_association_between_road_safety_and_socio-economic_situation_(SES).pdf. Accessed 19 Feb 2022

[CR34] Vingilis E, Mann RE, Erickson P, Toplak M, Kolla NJ, Seeley J, Jain U (2014). Attention deficit hyperactivity disorder, other mental health problems, substance use, and driving: examination of a population-based, representative Canadian sample. Traffic Inj Prev.

